# A case of inflammatory myofibroblastic tumor of the urinary bladder with emergency clinical symptoms similar to bladder cancer

**DOI:** 10.1016/j.eucr.2021.101740

**Published:** 2021-06-04

**Authors:** Yuki Matsui

**Affiliations:** Department of Urology, Showa University Fujigaoka Hospital, 1-30, Fujigaoka, Aoba-ku, Yokohama-shi, Kanagawa, 227-0043, Japan

**Keywords:** Bladder cancer, Bladder tamponade, Inflammatory myofibroblastic tumor, TURBT

## Abstract

A 55-year-old man was admitted for ongoing gross hematuria and bladder tamponade. Computed tomography revealed a mass near the right sidewall of the bladder, along with massive blood clots. The patient was diagnosed as having bladder cancer based on laboratory findings and emergency clinical symptoms. Thus, emergency transurethral resection of the bladder tumor was performed. Pathological examination revealed an inﬂammatory myoﬁbroblastic tumor (IMT). No tumor progression was observed during the 6-month follow-up period. Owing to its rarity, IMT has not been well characterized clinically and radiologically, and thus, it is very difficult to diagnose IMT accurately without pathological examinations.

## Introduction

Inﬂammatory myoﬁbroblastic tumor (IMT) of the bladder is a rare intermediate soft tissue tumor that is, composed of myoﬁbroblast-differentiated spindle cells accompanied by numerous inﬂammatory cells, plasma cells, and/or lymphocytes. IMT was first described in 1980 by Roth.^1^ Although IMT mainly occurs mainly in the lungs, it can also be found in the head and neck soft tissue, abdominal cavity, omentum, retroperitoneum, and other organs.^2^

The most common symptoms of IMT of the bladder include gross hematuria and dysuria.

It is believed that multiple causes such as urinary tract infection, prior history of surgery or instrumentation, diabetes mellitus, trauma, steroids, and immune disorders can lead to IMT development.^2^ However, the definitive pathogenesis of IMT is unclear and remains under discussion.

It is difficult to accurately diagnose such a rare disease based on findings of only imaging examinations and clinical symptoms. The ﬁnal identification of IMT often depends on histopathological features and immunohistochemical proﬁles.

According to the World Health Organization classification, IMT is designated as a tumor of intermediate biological potential owing to its low risk of distant metastasis.^2^

Surgical resection is the treatment of choice, and prognosis depends on local metastasis.^2^

Here, we report a case of IMT of the bladder that was clinically indistinguishable from urothelial carcinoma of the bladder.

## Case presentation

**Fig. 1:**
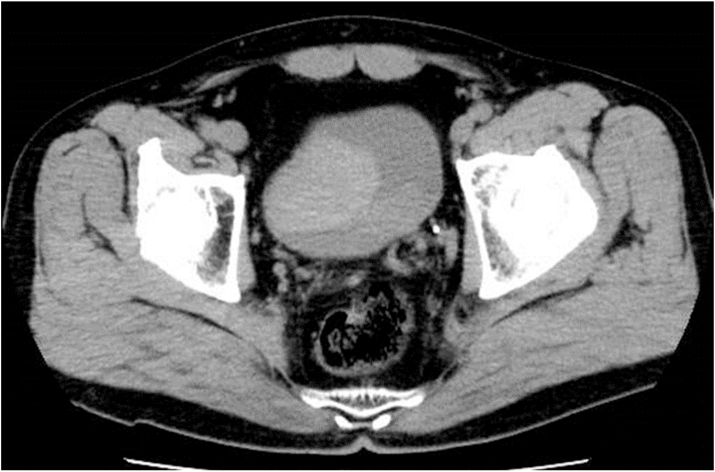
Computed tomography (CT) showed a mass near the bladder right sidewall, and massive blood clots in the bladder.

A 55-year-old man, with no signiﬁcant medical history, was admitted to the emergency room for ongoing gross hematuria with bladder tamponade. Blood examination results indicated a low hemoglobin level (6.2 g/L: 13.5–17.6g/L), and blood pressure was also low (78/60 mmHg). The patient was infused with two units of red blood cells (200 ml plasma transfusion) and also underwent continuous bladder irrigation to avoid catheter obstruction, which prevented blood clot formation. Abdominal and pelvic computed tomography scan revealed a mass near the right sidewall of the bladder and massive blood clots. (Fig. 1). Based on clinical symptoms and imaging findings, the patient was diagnosed as having bladder cancer, and underwent emergency transurethral resection of the bladder tumor (TURBT). His clinical symptoms improved, and he was discharged to home on the postoperative day.

**Fig. 2:**
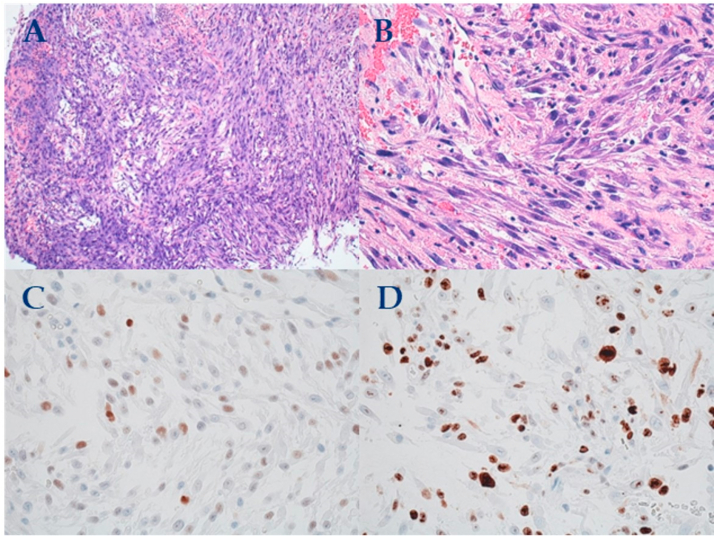
A,B：H&E staining show spindle myoepithelial cell proliferation and lymphocytic infiltrate ( × 200) C: ALK positive staining of spindle cells ( × 200) D: SMA positive staining of spindle cells ( × 200).

Pathological results showed the infiltration of a neoplasm, mainly composed of spindle cells with bland nuclear morphology and some with nuclear atypia arranged in fascicles in a vascular myxoid edematous background. A mixed population of inflammatory cells composed of lymphocytes, plasma cells, eosinophils, and neutrophils was present between neoplastic cells. Immunohistochemistry results were positive for vimentin, smooth muscle actin, Cytokeratin AE1/AE3, and anaplastic lymphoma kinase (ALK). (Fig. 2). Considering the sarcomatoid features and for excluding undifferentiated/sarcomatoid carcinoma, examed examination results desmin, epithelial membrane antigen, HHF35, and P63 were negative. The Ki67 proliferative activity was approximately 50%–70% in hotspot areas. Thus, the tumor was diagnosed as an IMT of the bladder. Subsequent follow-up cystoscopy was performed every 3 months after surgery. The first follow-up revealed no tumor recurrence or symptoms such as dysuria.

## Discussion

IMT is composed of myoﬁbroblast-differentiated spindle cells and is accompanied by numerous inﬂammatory cells, plasma cells, and lymphocytes. The most common site involved is the lungs, and the involvement of bladder is rare.^2^ A systematic review by Teoh et al. evaluated 182 cases of IMT of the bladder and reported that patients had a mean age of 38.9 years and that hematuria and dysuria were common clinical manifestations, with some patients also experiencing severe anemia.^3^ IMT of the bladder has a local tumor recurrence rate of only 4% after surgery, and only one patient with distant metastases has been reported.^2^ Since Hojo et al. showed an association between IMT and the ALK gene, many studies have reported the translocations of the ALK gene and expression of the ALK protein in IMTs with positive ALK immunostaining in 33%–89% of cases.^4^

To further study the IMT of the bladder, we reviewed relevant case reports published since 2010, including our own case (Table 1).^2^ The patient age ranged from 3 to 71 (mean 36.5) years, and females were represented more than males (ratio 15:11). Hematuria (n = 21) was a common clinical symptom in patients. The most common ﬁrst treatment option was TURBT (n = 19), followed by partial cystectomy (n = 10). There were five cases of emergency surgery owing to severe hematuria, and three cases had hypovolemic shock. ALK positivity was observed in 20 of the 26 cases.

**Table 1:** Summary of the clinical features and treatment of bladder of IMTs in the literature.

Summary of the clinical features and treatment of bladder of IMTs in the literature.							
Cases	Age	SEX	Clinical manifestations	Hemoglobin(g/L)	Tumor size(cm)	Management	ALK positive
1	3	M	Gross hematuria	unknown	unknown	Partial cystectomy	unknown
2	61	M	Gross hematuria	unknown	2	TURBT	Negative
3	58	M	Gross hematuria	unknown	3	TURBT Radical cystectomy	unknown
4	30	M	Gross hematuria	9	unknown	Partial cystectomy	Positive
5	38	F	Dysuria and pelvic pain	unknown	unknown	Urinary bladder transurethral resection	Positive
6	26	F	Hematuria, severe anemia	unknown	3.3	TURBT Radical cystectomy	Positive
7	40	M	Hematuria, dysuria, abdominal pain	unknown	5	TURBT	Positive
8	38	M	Burning micturition, Terminal macroscopic hematuria	unknown	3.2	Partial cystectomy	Positive
9	56	M	Hematuria	8.6	6	Urgent TURBT	Positive
10	17	F	Gross hematuria, lower abdominal pain	unknown	10	TURBT Partical cystectomy	Positive
11	29	M	Painless gross hematuria	unknown	4	Robot-assisted partial cystectomy	Positive
12	62	F	Visible hematuria	5.8	4	Urgent TURBT Partial cystectomy	Positive
13	71	F	Massive visible hematuria, Suprapubic pain, dysuria	8.6	3	TURBT	unknown
14	52	M	Gross hematuria	unknown	3	TURBT Partical cystectomy	Positive
15	36	M	Gross hematuria	unknown	4	TURBT	Positive
16	31	F	Abdominal pain, dysuria, nocturia, frequency, hematuria	unknown	2.3	Partial cystectomy	Positive
17	19	F	Hypogastrium pain	unknown	4	Partial cystectomy	Positive
18	23	F	Gross hematuria, hypovolemic shock	unknown	3	TURBT Partical cystectomy	Positive
19	31	F	Painful urination	unknown	4.5	TURBT	unknown
20	39	F	Severe hematuria	4.8	3.2	TURBT	Positive
21	40	F	difficult micturition, dyspareunia	unknown	4	TURBT	Positive
22	42	F	Gross hematuria	7	Unknown	TURBT、Radical cystectomy	Positive
23	11	F	Repeated urinary tract infection	unknown	7.8	Partical cystectomy	Negative
24	14	F	Gross hematuria	unknown	2	TURBT	Positive
25	28	F	Gross hematuria	6.2	4	TURBT	Positive
26	55	M	Gross hematuria	6.2	4	TURBT	Positive

Since IMT of the bladder is a rare tumor, it is difficult to suspect IMT based only on symptoms and imaging findings as it has not been well studied. The characteristics and causes of IMT of the bladder are expected to become further clear with the accumulation of more studies in the future.

## Conclusion

IMT of the bladder is a very rare tumor, and often presents with unpredictable clinical behavior. Thus, it requires complete surgical resection and regular monitoring of clinical outcomes.

We recommend clinical and radiological follow-up for monitoring the recurrence and metastasis of IMT of the bladder.

## Consent

Written informed consent was obtained from the participant for the publication of this case report. A copy of the written consent is available for the editorial review.

## Disclosure of sources of financial support

None.

## Declaration of competing interest

Authors have no conflict of interest to declare.
